# Proton density fat fraction of the spinal column: an MRI cadaver study

**DOI:** 10.1186/s12938-020-00846-4

**Published:** 2021-01-07

**Authors:** Merle S. Losch, Akash Swamy, Adrian Elmi-Terander, Erik Edström, Benno H. W. Hendriks, Jenny Dankelman

**Affiliations:** 1 Department of Biomechanical Engineering, Delft University of Technology, Delft, The Netherlands; 2 Department of In-Body Systems, Philips Research, Royal Philips, NV Eindhoven, The Netherlands; 3 Department of Clinical Neuroscience, Karolinska Institutet, Stockholm, Sweden; 4 Department of Neurosurgery, Karolinska University Hospital, Stockholm, Sweden

**Keywords:** Magnetic resonance imaging, Bone detection, Lipid content, Screw placement, Minimally invasive spine surgery

## Abstract

**Background:**

The increased popularity of minimally invasive spinal surgery calls for a revision of guidance techniques to prevent injuries of nearby neural and vascular structures. Lipid content has previously been proposed as a distinguishing criterion for different bone tissues to provide guidance along the interface of cancellous and cortical bone. This study aims to investigate how fat is distributed throughout the spinal column to confirm or refute the suitability of lipid content for guidance purposes.

**Results:**

Proton density fat fraction (PDFF) was assessed over all vertebral levels for six human cadavers between 53 and 92 years of age, based on fat and water MR images. According to their distance to the vertebra contour, the data points were grouped in five regions of interest (ROIs): cortical bone (−1 mm to 0 mm), pre-cortical zone (PCZ) 1–3 (0–1 mm; 1–2 mm; 2–3 mm), and cancellous bone ($\ge$ 3 mm). For PCZ1 vs. PCZ2, a significant difference in mean PDFF of between −7.59 pp and −4.39 pp on average was found. For cortical bone vs. PCZ1, a significant difference in mean PDFF of between −27.09 pp and −18.96 pp on average was found.

**Conclusion:**

A relationship between distance from the cortical bone boundary and lipid content could be established, paving the way for guidance techniques based on fat fraction detection for spinal surgery.

## Background

Spinal fusion surgery is performed to treat fractures, reduce back pain or correct for spinal deformities due to scoliosis or degenerative spine conditions [1–3]. The vertebrae are typically fixed to each other with the use of metal rods anchored to the bone through pedicle screws. Pedicle screws run through the pedicle while their heads provide dedicated attachment points for the rods. However, the relatively soft tissue on the inside of the bone, the cancellous bone, is not strong enough for spine fixation. Therefore, pedicle screw fixation mainly relies on locations where the screw is in direct contact with the surrounding bone layer, the dense cortical bone [4]. Increasing the contact area between screws and cortical bone is thought to result in better fixation of the pedicle screws [5–7].

Spinal fusion is commonly carried out in a minimally invasive surgery (MIS) procedure as this shortens length of hospital stay and recovery [8, 9]. MIS is performed through several small incisions, which necessitates guidance techniques for the surgeon to compensate for the limited visibility of the surgical site. The close proximity of neural and vascular structures and the inability to adjust the trajectory of the pedicle screw after insertion present an additional challenge to screw placement [10, 11].

One possible approach to maximize the contact area between screws and cortical bone is to replace the conventionally used straight screws with a flexible anchoring device that runs along the interface of cancellous and cortical bone on a curved trajectory close to the outer edge of the vertebra. The need for reliable guidance is evident for such a device. Hence, it is crucial to determine reliable distinguishing criteria for cancellous and cortical bone that help to identify the correct trajectory. Burström et al. [12] have suggested lipid content as such a criterion and shown its potential to predict impending breaches in pedicle screw placement.

Lipids in the human body are commonly quantified using MRI. Several research groups have used this medical imaging technique to non-invasively measure fat fraction in the vertebral body to evaluate various clinical conditions such as osteoporosis [13, 14], cancer [15], and metabolic disorders such as obesity and diabetes [16, 17]. However, few studies focusing on the distribution of fat fraction within the vertebrae for distinction of cancellous and cortical bone are available so far [18].

This study, therefore, aims to investigate the fat fraction distribution throughout the spinal column of human cadavers and to identify a possible relationship between the distance from the cortical bone boundary and lipid content. The transition area between cancellous and cortical bone is of particular interest, given that a flexible anchoring device would be located in this area.

## Results

### Extracted PDFF data

For the cortical bone and the three PCZs, between 300 and 3 500 individual PDFF values per vertebra and cadaver were extracted, respectively, depending on the vertebra size. The ROI of cancellous bone contained up to 20 000 data points for the largest vertebrae.

**Fig. 1:**
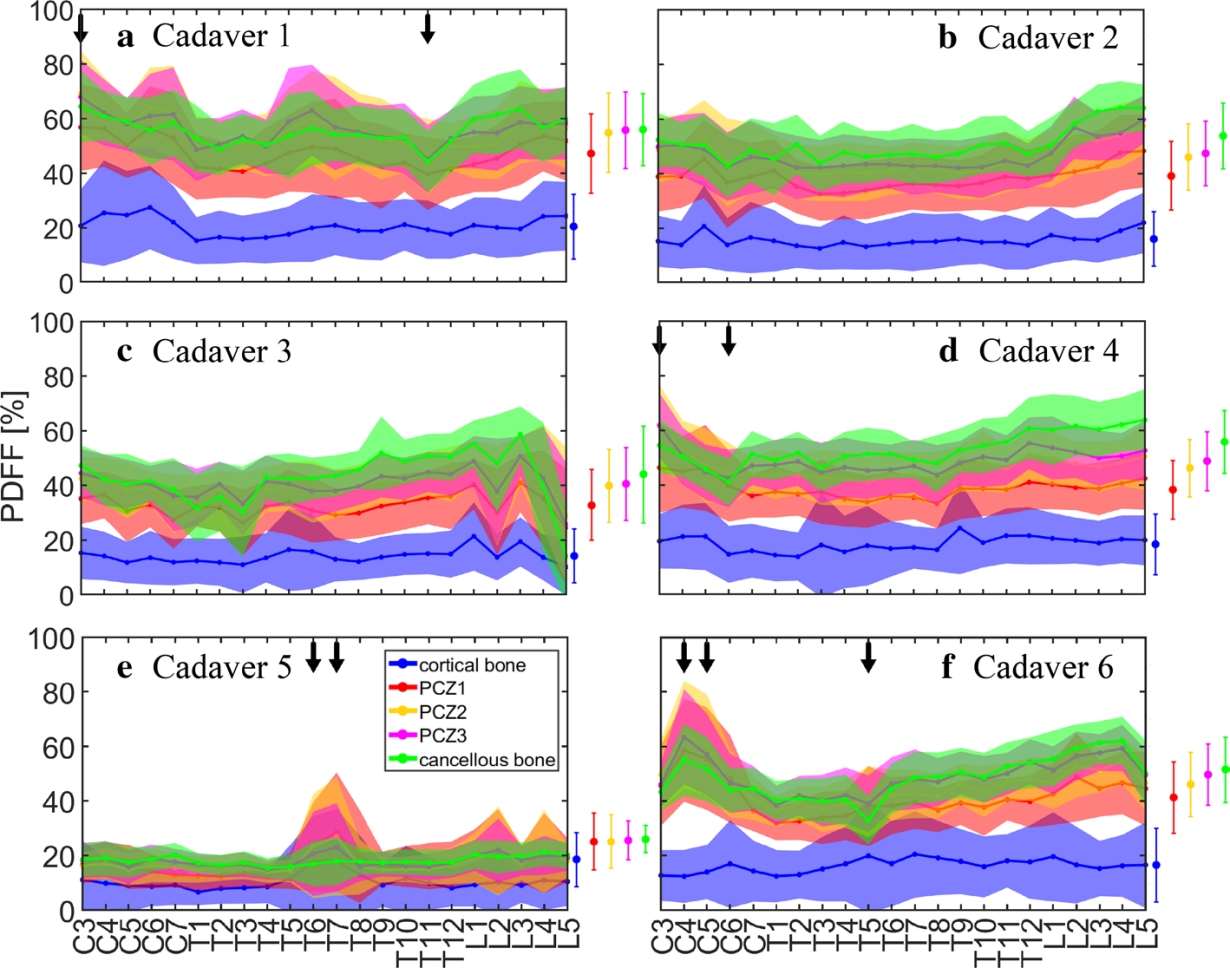
PDFF distributions over all vertebral levels and whole spine mean PDFFs and standard deviations

Figure 1 displays the PDFF distributions obtained for the individual ROIs over all vertebral levels. The mean PDFF is displayed as a solid line. To each side, one standard deviation is highlighted in the corresponding color. The mean PDFFs and standard deviations over the whole spine are displayed to the right of each plot for the ROIs of the corresponding cadaver.

Figure 1 shows similar PDFF distributions for all cadavers, with the exception of Cadaver 5, which shows very low overall PDFF in comparison to the other cadavers.

The PDFF distributions show that natural variation among the individual vertebrae exists. This variation becomes especially apparent in Cadaver 3 (Fig. 1c). For the other cadavers, however, large deviations from the whole spine mean PDFF are rare and only occur for singular vertebrae (marked in Fig. 1 with downward pointing arrows).

All cadavers but Cadaver 5 exhibit a mean cortical bone PDFF of around 15–20%. The curve of the cortical bone PDFF distribution is distinct from the distribution curves of the other ROIs. Mean PCZ1 PDFF lies between 30 and 50%. For each of the cadavers, the PCZ1 PDFF distribution curve is partly below the distribution curves of the remaining ROIs. PCZ2, PCZ3, and cancellous bone exhibit elevated mean PDFF values, and their PDFF distribution curves overlap to a large extent.

In Cadaver 5, the mean cortical bone PDFF is around 10%. The PDFF distribution curves for PCZ1, PCZ2, PCZ3 and cancellous bone overlap and exhibit mean values below 20%.

### Results of the statistical analysis

**Fig. 2:**
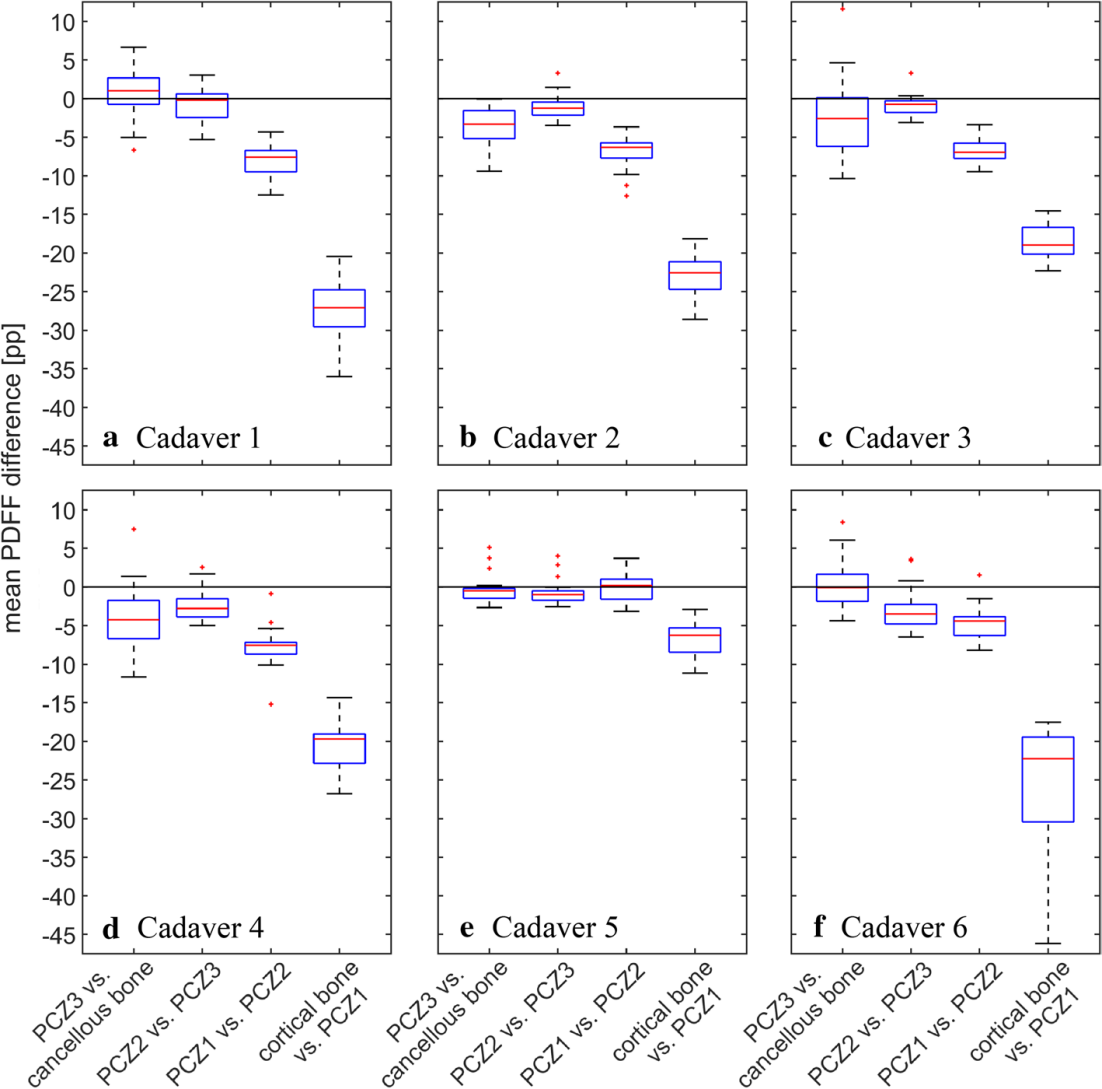
Mean PDFF differences for all vertebrae

The box plots in Fig. 2 display the distribution of the mean differences in the PDFF for adjacent ROIs observed for the individual vertebrae of each cadaver. The plots show that mean differences behave similarly in all cadavers, with the exception of Cadaver 5.

All cadavers but Cadaver 5 exhibit mean PDFF differences for PCZ3 vs. cancellous bone and for PCZ2 vs. PCZ3 whose plots are around or contain the value zero. The plots for PCZ1 vs. PCZ2 are below zero and display median values between −7.59 percentage points (pp) (Cadaver 1) and −4.39 pp (Cadaver 6). The plots for cortical bone vs. PCZ1 are also below zero and display median values between −27.09 pp (Cadaver 1) and −18.96 pp (Cadaver 3).

When used for guidance in spinal fusion surgery, fat fraction detection should enable the surgeon to detect the bone boundary and prevent breaching it. Assuming the surgeon to approach the outer layer (PCZ and cortical bone) from the inside of the bone, the first observed mean difference is at the interface of cancellous bone and PCZ3. After excluding the outliers, this difference is between −11.64 pp (Cadaver 4) and +6.66 pp (Cadaver 1) for the examined samples. For the transition from PCZ3 towards PCZ2, the mean PDFF difference varies from −6.48 pp (Cadaver 6) to +3.02 pp (Cadaver 1).

A difference in the mean PDFF of between −12.48 pp (Cadaver 1) and −1.53 pp (Cadaver 6) is found from PCZ2 towards PCZ1. The mean PDFF in the cortical bone changes by between −46.19 pp (Cadaver 6) and −14.34 pp (Cadaver 4) as compared to PCZ1.

For Cadaver 5, the plots of the mean PDFF differences for PCZ3 vs. cancellous bone, for PCZ2 vs. PCZ3 and for PCZ1 vs. PCZ2 all contain the value zero. The plot for cortical bone vs. PCZ1 is below zero and displays a median value of −6.25 pp.

## Discussion

In this study, we have investigated the distribution of PDFF in human cadaver vertebrae, with a particular focus on the transition zone between the cancellous and cortical regions of the bone (PCZ). As a flexible anchoring device would be affixed in the PCZ along the outer edge of the vertebra, the area around the spinal cord was considered irrelevant for the given application and was thus not part of the analysis.

The cadavers included in this study had a mean age of 77.8 years and belonged to the patient cohort of older adults, which is the most common cohort for spinal fusion surgery. Patients in this cohort may suffer from back pain due to various clinical conditions including degenerative disk disease and spinal stenosis [2]. Measurements of PDFF in the cancellous bone from this ex-vivo study are in line with the results of previous in vivo research on similar patient cohorts [13, 19, 20].

Similar PDFF distributions and mean PDFFs were observed for all cadavers, except for Cadaver 5. The study results show lower PDFF for Cadaver 5 compared to the rest of the cadavers studied. This subject was found to suffer from malignant neoplasm of the esophagus, which may be the cause of the low PDFF found across all spinal levels. Patients with active malignancy have a higher chance of perioperative complications and are less likely to be considered for spinal fusion surgery [21, 22]. The PDFF measurements for Cadaver 5 can therefore be assumed to be non-representative of vertebral body fat fraction in spinal surgery patients. The mean PDFF difference between cortical bone and PCZ1 found for this cadaver suggests that guidance based on fat fraction may still be possible for patients with active malignancies, but parameters would have to be assessed separately for these patients.

For the other cadavers, the observed PDFF distributions suggest that cortical bone can be distinguished from the remaining ROIs. Fat fraction seems to increase gradually from cortical bone through PCZ1 up to the three innermost ROIs (PCZ2, PCZ3, and cancellous bone). As the PDFF distributions of these three ROIs overlap, no distinction based on PDFF measurements seems possible here.

Statistical analysis confirms these findings: when examining the mean PDFF difference of PCZ3 vs. cancellous bone, no significance is found, as both positive and negative values are observed. Equally, for the mean PDFF difference of PCZ2 vs. PCZ3, the observed values do not consistently have the same sign, hence these zones are not considered significantly different.

When advancing from PCZ2 towards PCZ1, a first significant drop in the mean PDFF can be observed. For the examined samples, the average difference was between −7.59 pp (Fig. 2a) and −4.39 pp (Fig. 2f). Although consistently negative, the absolute values of the observed differences are small for some vertebrae, and it needs to be verified whether they can reliably serve for guidance in spinal fusion surgery.

When further advancing from PCZ1 towards cortical bone, another significant decrease in the mean PDFF is found. For the examined samples, the average difference was between −27.09 pp (Fig. 2a) and −18.96 pp (Fig. 2c). This decrease is in the same order of magnitude as the total mean cortical bone PDFF, and can, therefore, very likely be detected intra-operatively, and thus prevent the surgeon from traversing the cortical bone boundary.

For singular vertebrae, the PDFF curves reveal unusually large deviations from the whole spine mean. These vertebrae also show an altered anatomy on the PDFF MR images. Modic changes that come along with degenerative edema can lead to elevated grayscale values, which are associated with a high PDFF [23]. Another cause for large deviations from the whole spine mean are sclerotic lesions, which can, for instance, manifest as bone islands—intramedullary condensations of cortical bone which appear as areas with low signal intensity.

In Cadaver 3, which reveals a particularly high variation in PDFF (Fig. 1c), several vertebrae exhibit dark spots. Possible explanations are an underlying malignancy with metastases that have destroyed the bone partially, or posterior vertebral scalloping that is a possible result of a variety of pathologies such as degenerative spine conditions, dural ectasia, and intraspinal tumors deforming the vertebra [24]. The influence of such anomalies on lipid content in the vertebrae needs to be researched, although the mean differences acquired in this study do not reveal substantial discrepancies for vertebrae with an altered anatomy.

Furthermore, it has been shown previously that the PDFF changes over the course of a lifetime [25]. This study focused on the most common patient cohort of older adults, and none of the examined cadavers belonged to the other patient cohort of adolescents suffering from spinal deformities [1]. A further study investigating fat fraction distribution in the vertebrae of this patient cohort is encouraged.

It could be argued that the vertebral fat content of cadavers may not represent the in vivo fat content due to postmortem changes. However, a study by Lamoureux et al. [26] showed that bovine and equine percentage of fat in bone marrow does not change within 30–60 days after necropsy, regardless of the storage condition. In an in vivo human study by de Boer et al. [27] fat content was assessed on tissue samples both before and after resection. Comparison of the measurements did not yield any significant differences.

### Limitations

The model used for water–fat separation assumes that objects are scanned at body temperature. Although this model is relatively stable to variations in temperature, the calculated PDFF values might be slightly biased, as the cadavers examined in this study were not scanned at body temperature but at room temperature.

Selection of the vertebra contours was done by manually detecting high grayscale values on the MR image. Although the process was kept consistent for the entire dataset, it is prone to bias. Using CT images as ground truth for vertebra contour detection is recommended for future studies. Another possible approach to mitigate the bias is to increase the magnetic field strength from 1.5 T to 3 T for better separation of fat and water, thereby creating a higher contrast between cortical and cancellous bone on the MR images [28]. Image acquisition with an increased in-plane resolution could decrease the pixel size and thus increase the number of data points for each ROI.

The cortical bone ROI was grown automatically based on the detected vertebra contour and the assumption of a uniform cortical thickness of 1 mm. Swamy et al.[29] have shown cortical bone thickness to vary between 1 and 3 mm. However, cortical bone at a distance of more than −1 mm from the vertebra contour is expected to show an equal or lower PDFF compared to the cortical bone ROI as defined in this study, creating an even larger mean difference between cortical bone and PCZ1.

Lastly, investigating PDFF distributions across additional slices and other 3D planes could provide further insights, especially concerning the PDFF distribution in the pedicle area, a crucial region for screw placement.

## Conclusion

This study investigated the fat fraction distribution, quantified through MRI, throughout the spinal column of six human cadavers. Lipid content was found to be related to the distance from the cortical bone boundary, and significant mean PDFF differences between cortical bone and the PCZ were found. Hence, in this study, fat fraction is found to be a valid criterion for distinction between the different bone tissues in vertebrae and has the potential to provide guidance in spinal fusion surgery.

## Methods

In this research, six human cadavers (four females, two males) with an age range of 53–92 years (mean = 77.8 years) were studied. One of the subjects examined (Cadaver 5) was known to have suffered from malignant neoplasm of the esophagus. All cadavers were donated for scientific research. Informed consent had been signed before death by the donors or after death by relatives, according to local guidelines and U.S. regulations. The study was conducted in compliance with ethical guidelines for human cadaver studies.

### Image acquisition

#### MRI

The cadavers’ entire spines were scanned on a 1.5 Tesla (T) whole-body scanner (Ingenia, Philips Healthcare, Best, The Netherlands) in the prone position. The temperature of the cadavers was maintained at room temperature prior to scanning.

A three-dimensional (3D) six-echo spoiled gradient-echo sequence was used for chemical shift-encoding-based water–fat separation. The typical imaging parameters used in this study were: AP field of view = 220–310 mm; FH field of view = 240–350 mm; slice thickness = 3 mm; in-plane resolution = 1.2 $\times$ 1.2 $\hbox {mm}^2$; flip angle = $5^{\circ }$; TR = 9.9–15.87 ms; TE1 = 1.41–1.43 ms; $\Delta \hbox {TE}$ = 1.2 ms. Reconstruction with a voxel size of (0.45-0.67) $\times$ (0.45-0.67) $\times$ 1.5 $\hbox {mm}^3$ yielded 45–65 sagittal slices per sequence.

To obtain whole spine coverage, the MR exam consisted of three 3D spoiled gradient-echo sequences placed on the cervical, thoracic and lumbar spine, respectively. Total scan time per cadaver was approximately 5–10 min.

#### Determination of PDFF

The scanner image reconstruction was used to separate the signals of water and fat using Philips DICOM viewer R3.0-SP04 (Philips Healthcare, Best, The Netherlands). The water–fat separation was based on a seven-peak water–fat spectral model [30].

Based on fat signal (F) and water signal (W), proton density fat fraction (PDFF) was calculated as:

$$\begin{aligned} PDFF \ [\%] = \frac{F}{F+W}\cdot 100 \end{aligned}$$

**Fig. 3:**
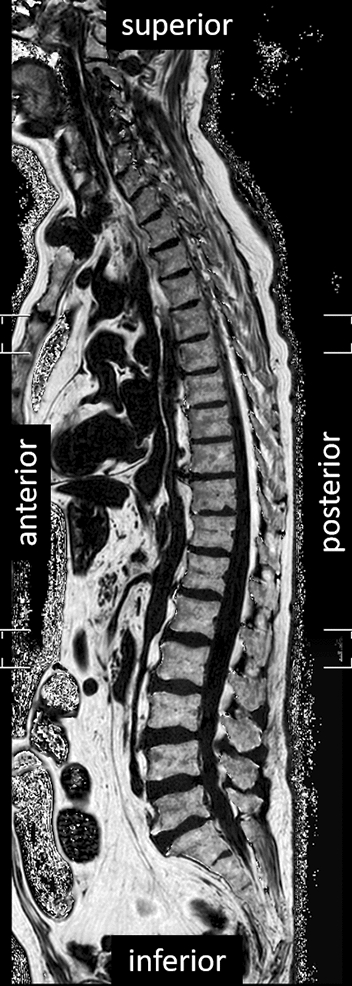
Mid-sagittal PDFF MR image of the whole spine (Cadaver 6)

Figure 3 shows the visual representation of a PDFF MR image of the mid-sagittal slice of the whole spine.

### Image analysis

For each cadaver, the three mid-sagittal slices (cervical, thoracic, lumbar) plus three respective adjacent slices on each side were retained for the analysis. The image analysis procedure was performed for vertebrae C3 through L5.

#### ROI selection

**Fig. 4:**
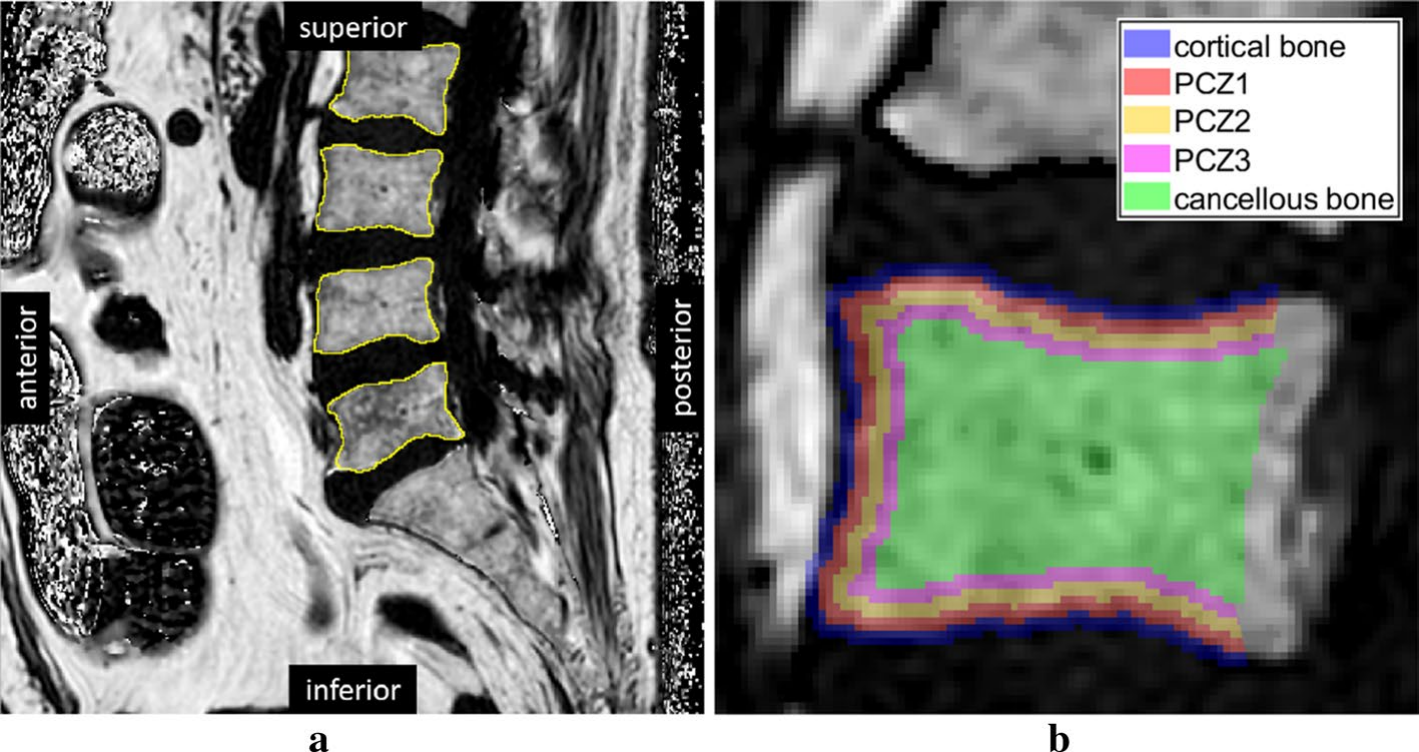
ROI selection process: **a** Mid-sagittal PDFF MR image of the lumbar spine with detected vertebra contours **b** Definition of the anatomical ROIs: cortical bone, pre-cortical zone (PCZ), and cancellous bone

For each slice, regions of interest (ROIs) were selected on the MR image using the FIJI distribution of ImageJ [31]. The contours of the single vertebrae were found based on grayscale value of the PDFF MR image and estimated vertebra shape, as shown in Fig. 4a. Anterior, inferior and superior walls were included in the analysis.

Burström et al.[12] have introduced the concept of a 3-mm-thick transition zone between cancellous and cortical bone, the so-called pre-cortical zone (PCZ). This idea was adopted in this work: pixels within a 3-mm distance from the vertebra contour were assigned to one of three adjacent pre-cortical ROIs of 1 mm thickness each (PCZ1, PCZ2, PCZ3). An ROI within the vertebra at a distance of more than 3 mm to the contour was considered to contain the cancellous bone. An ROI of cortical bone was defined as the first mm (−1 mm to 0 mm) outside the detected contour. The selected ROIs are shown in Fig. 4b. An overview of the ROIs can be found in Table 1.

**Table 1:** Overview of the defined ROIs

	Bone type	Distance from contour
ROI0	Cortical bone	−1 mm to 0 mm
ROI1	PCZ1	0 mm to 1 mm
ROI2	PCZ2	1 mm to 2 mm
ROI3	PCZ3	2 mm to 3 mm
ROI4	Cancellous bone	$\ge$ 3 mm

#### Data extraction

Fat fraction distributions were calculated for the ROIs listed in Table 1 by calculating the PDFFs for all pixels included in the respective ROI using MATLAB R2019b (The MathWorks Inc., Natick (MA), USA). For each vertebra within each cadaver, the data points gathered from the different slices were merged to yield one dataset per ROI.

### Statistical analysis

To provide guidance in the context of spinal fusion surgery, individual variation in fat fraction is considered most important, so the data were analyzed separately for every vertebra in each of the cadavers.

Mean differences of the measured PDFFs were computed for the respective adjacent ROIs (cortical bone vs. PCZ1; PCZ1 vs. PCZ2; PCZ2 vs. PCZ3; PCZ3 vs. cancellous bone) as the difference of the outer ROI’s mean PDFF value to the inner ROI’s mean PDFF value. As reliable guidance is crucial for spinal surgery, a pair of adjacent ROIs was only considered significantly different if, after the exclusion of potential outliers, the mean differences observed for this pair consistently had the same sign for *all* vertebrae.

## Data Availability

The MRI datasets analyzed during the current study were collected by Philips following the required regulatory approval and are therefore not publicly available. The processed data is available at 10.4121/13089956.

## Abbreviations

3D: Three-dimensional
MIS: Minimally invasive surgery
PCZ: Pre-cortical zone
PDFF: Proton density fat fraction
pp: Percentage points
ROI: Region of interest
